# External Validation of Risk Scores for Major Bleeding in a Population-Based Cohort of Transient Ischemic Attack and Ischemic Stroke Patients

**DOI:** 10.1161/STROKEAHA.117.019259

**Published:** 2018-02-19

**Authors:** Nina A. Hilkens, Linxin Li, Peter M. Rothwell, Ale Algra, Jacoba P. Greving

**Affiliations:** From the Julius Center for Health Sciences and Primary Care (N.A.H., A.A., J.P.G.) and Department of Neurology and Neurosurgery, Brain Center Rudolf Magnus (A.A.), University Medical Center Utrecht, Utrecht University, the Netherlands; and Nuffield Department of Clinical Neurosciences, University of Oxford, United Kingdom (L.L., P.M.R.).

**Keywords:** antiplatelet agents, bleeding, human, risk, stroke

## Abstract

**Background and Purpose—:**

The S_2_TOP-BLEED score may help to identify patients at high risk of bleeding on antiplatelet drugs after a transient ischemic attack or ischemic stroke. The score was derived on trial populations, and its performance in a real-world setting is unknown. We aimed to externally validate the S_2_TOP-BLEED score for major bleeding in a population-based cohort and to compare its performance with other risk scores for bleeding.

**Methods—:**

We studied risk of bleeding in 2072 patients with a transient ischemic attack or ischemic stroke on antiplatelet agents in the population-based OXVASC (Oxford Vascular Study) according to 3 scores: S_2_TOP-BLEED, REACH, and Intracranial-B_2_LEED_3_S. Performance was assessed with C statistics and calibration plots.

**Results—:**

During 8302 patient-years of follow-up, 117 patients had a major bleed. The S_2_TOP-BLEED score showed a C statistic of 0.69 (95% confidence interval [CI], 0.64–0.73) and accurate calibration for 3-year risk of major bleeding. The S_2_TOP-BLEED score was much more predictive of fatal bleeding than nonmajor bleeding (C statistics 0.77; 95% CI, 0.69–0.85 and 0.50; 95% CI, 0.44–0.58). The REACH score had a C statistic of 0.63 (95% CI, 0.58–0.69) for major bleeding and the Intracranial-B_2_LEED_3_S score a C statistic of 0.60 (95% CI, 0.51–0.70) for intracranial bleeding. The ratio of ischemic events versus bleeds decreased across risk groups of bleeding from 6.6:1 in the low-risk group to 1.8:1 in the high-risk group.

**Conclusions—:**

The S_2_TOP-BLEED score shows modest performance in a population-based cohort of patients with a transient ischemic attack or ischemic stroke. Although bleeding risks were associated with risks of ischemic events, risk stratification may still be useful to identify a subgroup of patients at particularly high risk of bleeding, in whom preventive measures are indicated.

## Abstract 2

Supplemental Digital Content is available in the text.

**See related article, p 513**

Lifelong secondary prevention with antiplatelet agents is recommended in patients who experienced a transient ischemic attack (TIA) or ischemic stroke.^1^ Bleeding is a clinically important and potentially life-threatening side effect of antiplatelet drugs.^2^ Risk of bleeding increases steadily with age, and the gastrointestinal (GI) tract is shown to be the most common source of bleeding.^3–5^ Individualized prediction of bleeding risk may help physicians to identify patients at highest risk and may guide treatment decisions regarding initiation of gastroprotective agents.

Recently, the S_2_TOP-BLEED score was developed to predict risk of major bleeding in patients with a TIA or ischemic stroke on antiplatelet agents.^6^ The model was derived from individual patient data from 6 randomized clinical trials (Table I in the online-only Data Supplement),^7–12^ including over 43 000 patients with a TIA or ischemic stroke, and was subsequently validated in the PERFORM trial (Prevention of Cerebrovascular and Cardiovascular Events of Ischaemic Origin With Terutroban in patients With a History of Ischaemic Stroke or Transient Ischaemic Attack Study),^13^ including another 19 000 patients with a recent TIA or ischemic stroke.

A potential drawback of using trial data for development of a risk score is that participants may represent a selective subset of the population of interest, as frail and elderly patients are often excluded from trials. As a consequence, absolute risks may be underestimated in a real-world setting and associations between predictors and outcome may differ.^14,15^ External validation of a risk score in observational data could, therefore, provide valuable insight into the accuracy of the predicted risks and the generalizability to a wider range of patients. We aimed to externally validate the S_2_TOP-BLEED score in a population-based cohort and to assess its performance according to site and severity of bleeding. Subsequently, we compared its performance to other risk scores for bleeding in patients with a TIA or ischemic stroke.

## Methods

### Study Population

The OXVASC (Oxford Vascular Study) is an ongoing population-based study on the incidence and outcome of all acute vascular events in Oxfordshire, United Kingdom. Methods and definition of events have been described previously.^16^ Briefly, the study population comprises 92 728 individuals, registered with 100 general practitioners in 9 general practices in Oxfordshire. Multiple overlapping methods of hot and cold pursuit are used for ascertainment of all acute vascular events in the study population, which has been shown to be near complete.^17^ For the current analysis, we studied patients with a TIA or ischemic stroke between 2002 and 2012, who were on antiplatelet drugs after their event. These included both patients who were on premorbid antiplatelet drugs, as well as patients who started antiplatelet drugs after the index event. Patients who switched to oral anticoagulants during follow-up were censored at the time of starting (Table I in the online-only Data Supplement).

Information on patient demographics and vascular risk factors was collected during the initial assessment. Patients were followed-up face to face by a study nurse or physician at 1 month, 6 months, and 1, 5, and 10 years after the index event. Recurrent ischemic events, bleeding events that required medical attention, and disability (modified Rankin Scale) were recorded at each follow-up. Bleeding events were also identified by daily searches of all hospital admissions, by review of administrative diagnostic codes from hospital and primary care records, and by searches of blood transfusion records. Only bleeds that required medical attention or were fatal prior to medical attention could be sought were included. Bleeds secondary to trauma, surgery, or hematological malignancy were excluded.

Bleeds were classified according to site of hemorrhage as either intracranial (intracerebral, subarachnoid, and subdural), upper GI, lower GI, epistaxis, genitourinary, or other. The severity of bleeds was recorded according to the CURE criteria (Clopidogrel in Unstable Angina to Prevent Recurrent Events).^18^ Major bleeds were bleeds that were substantially disabling with persistent sequelae, intraocular bleeds leading to significant loss of vision, or bleeds requiring transfusion of ≥2 units of blood. Major bleeds were classified as life-threatening if the bleeding episode was fatal, symptomatic intracranial, led to a reduction in hemoglobin level of at least 5 g/dL (3.1 mmol/L), led to substantial hypotension requiring use of intravenous inotropic agents, necessitated a surgical intervention, or necessitated transfusion of ≥4 units of blood. Bleeding events that required medical attention but did not fulfill the criteria of major bleeding were recorded as significant nonmajor bleeds. OXVASC has been approved by the local ethics committee, and all participants gave written informed consent. Requests for anonymized data will be considered by Professor Rothwell (peter.rothwell@clneuro.ox.ac.uk).

### Statistical Analysis

Data were missing on body mass index in 79 patients (4%) and on smoking in 3 patients (<1%). These patients were excluded from the analysis. Variables of the S_2_TOP-BLEED score (Table II in the online-only Data Supplement) were matched to variables in OXVASC. A proxy was used if no direct match was available. The National Institutes of Health Stroke Scale was used to assess severity of the index event and was used as a proxy for the modified Rankin Scale score, where a National Institutes of Health Stroke Scale score ≤3 was considered a minor stroke and a score >3 a severe stroke. All patients who received a short course of aspirin plus clopidogrel (for the first 30–90 days) and were treated with aspirin (plus dipyridamole) thereafter were analyzed as if they were on aspirin (plus dipyridamole), as our interest was in long term risk of bleeding.

The original regression equation was applied to the validation data to calculate 3-year risk of major bleeding. We assessed discriminatory performance of the model with the C statistic and calibration with the calibration slope and plots. Calibration at 3 years was examined by dividing patients in quintiles according to their predicted risk. The mean predicted risk per quintile group was subsequently plotted against the observed risk per quintile group. Calibration over time was assessed across risk groups that were predefined as low risk (0–10 points on the S_2_TOP-BLEED score), medium risk (11–15 points), and high risk (>15 points).^6^ Model performance was also assessed separately by severity of bleeding (nonmajor, major and life-threatening, or fatal) and by site of bleeding (intracranial, upper GI, lower GI, epistaxis, genitourinary, or other). We performed a sensitivity analysis excluding patients with an established high risk of bleeding or reduced life expectancy (patients with renal failure, liver failure, cancer, or a prior peptic ulcer) who are generally not included in trials.

We compared performance of the S_2_TOP-BLEED score with performance of the REACH score for major bleeding,^19^ and the Intracranial-B_2_LEED_3_S score (low BMI, high blood pressure, lacune, elderly, Asian ethnicity, cardiovascular disease, cerebrovascular disease, dual antithrombotic treatment or anticoagulant, sex) for intracranial hemorrhage after TIA or ischemic stroke (Table III in the online-only Data Supplement),^20^ by means of the C statistic, integrated discrimination improvement, and net reclassification improvement.^21,22^ Another risk score for intracranial hemorrhage after TIA or stroke could not be validated as it required postacute blood glucose levels, which were not available in the validation cohort. To study the influence of the different age categories used in the different risk scores for major bleeding on the performance, we assessed the C statistic of the models containing age only and compared it to the C statistic of the remainder of the model.

As risk factors for bleeding events are also known to be risk factors for recurrent ischemic events, we assessed the discriminatory ability of the S_2_TOP-BLEED score for recurrent ischemic events at 3 years (defined as recurrent ischemic stroke, myocardial infarction, or sudden cardiac death). Next, we assessed the cumulative incidence of bleeding events and recurrent ischemic events at 3 years and their ratio across risk groups of the S_2_TOP-BLEED score. Results are reported in accordance with the TRIPOD statement (Transparent Reporting of a Multivariable Prediction Model for Individual Prognosis or Diagnosis).^23^ All analyses were performed with R version 3.3.2.

## Results

**Figure 1.**
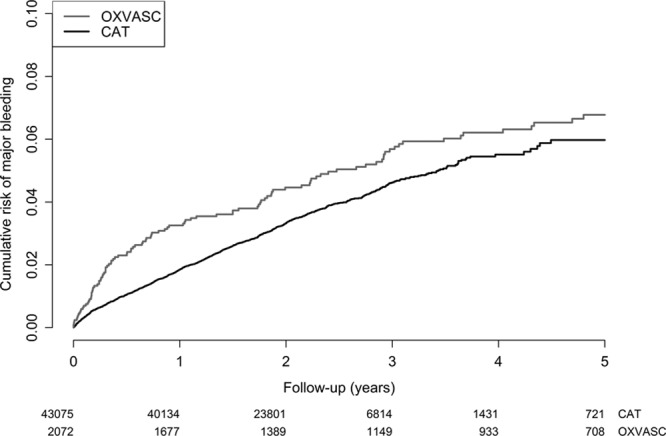
Cumulative risk of major bleeding in development cohort (CAT) and validation cohort (OXVASC). CAT indicates Cerebrovascular Antiplatelet Trialists; and OXVASC, Oxford Vascular Study.

Between 2002 and 2012, 2072 patients with a TIA or ischemic stroke on antiplatelet drugs were included in OXVASC. Median follow-up was 3.5 years (interquartile range 1.5–6.3). Baseline characteristics of patients in the development and validation cohort are shown in Table 1. Patients in OXVASC were older than patients in the CAT trials (Cerebrovascular Antiplatelet Trialists; mean age 73 years [SD, 13.4] versus 66 years [SD 9.7]). Two hundred fifty-four bleeds occurred during follow-up, of which 117 (46%) were major bleeds. Upper GI bleeds were the most common type of bleeding (32%; Table IV in the online-only Data Supplement). Four hundred sixty-one patients (22%) were classified as having an established high risk of bleeding, and 39% of all major bleeds occurred within this group. Risk of major bleeding was higher in the validation cohort than in the development cohort (Figure 1).

**Table 1.**
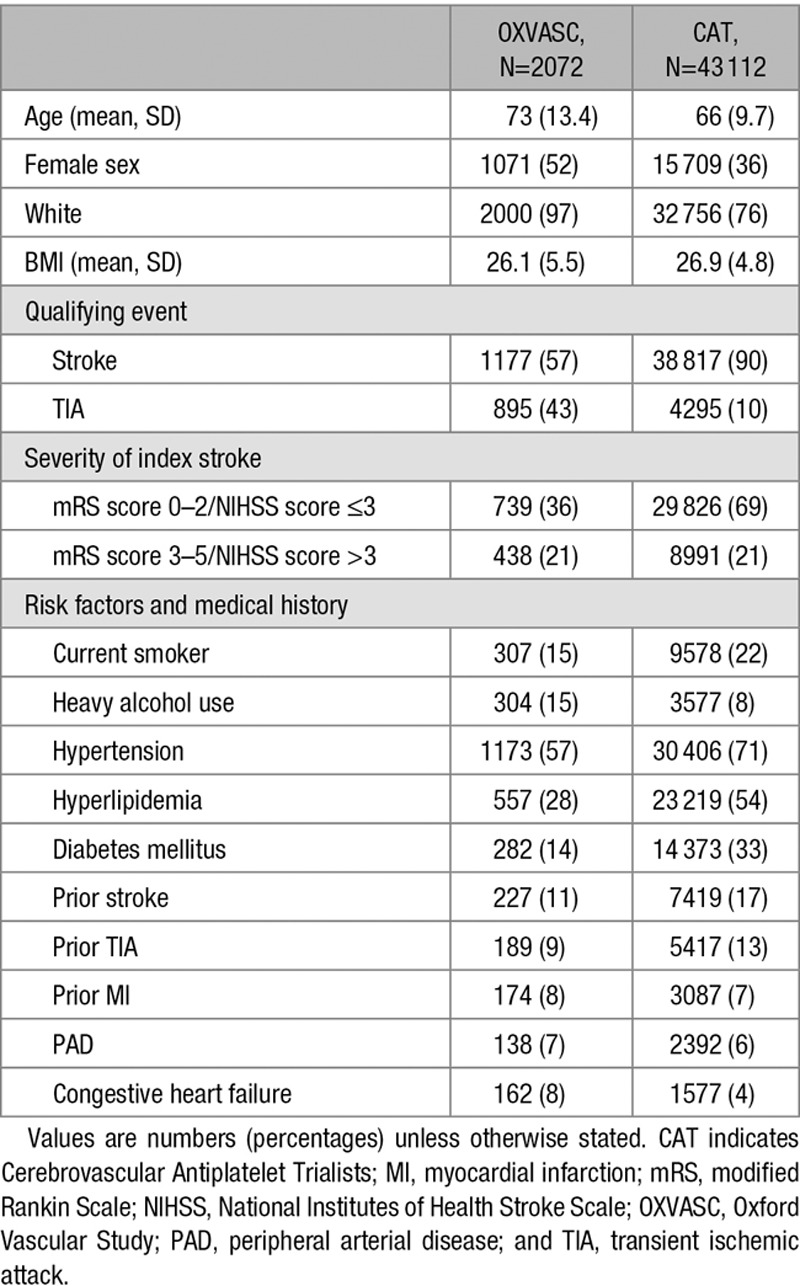
Baseline Characteristics of Patients in Development (CAT) and Validation Cohort (OXVASC)

**Figure 2.**
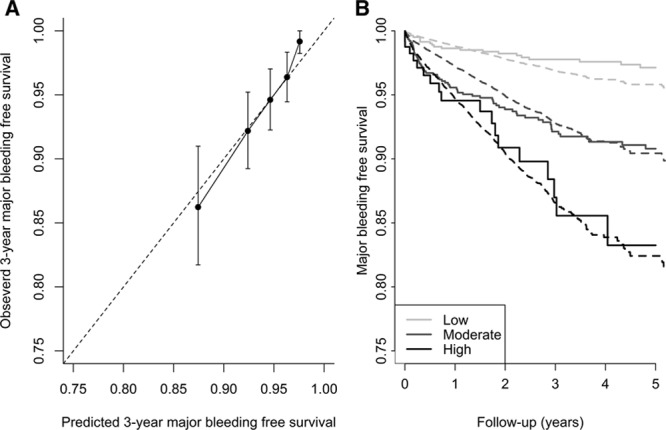
Calibration plots for the S2TOP-BLEED score. Calibration plots: 3-year major bleeding-free survival (**A**) and calibration across risk groups (**B**). Correspondence between observed and predicted 3-year major bleeding-free survival across quintile groups (**A**). Observed risk (solid line) and predicted risk (dotted line) across predefined risk groups of the S_2_TOP-BLEED score (**B**).

The C statistic of the S_2_TOP-BLEED score for major bleeding was 0.69 (95% confidence interval [CI], 0.64–0.73) and calibration at 3 years was accurate (calibration slope 1.13, *P*=0.48; Figure 2A). Early risk of bleeding was underestimated by the model, but calibration across risk groups was accurate for long term risk of bleeding (Figure 2B). The S_2_TOP-BLEED score was much more predictive for fatal and major bleeding (C statistic 0.77 and 0.69) than for nonmajor bleeds (C statistic 0.50; Table 2). Discriminatory ability was higher for intracranial and upper GI bleeds than for lower GI bleeds, genitourinary bleeds, and epistaxis (Table 2). A sensitivity analysis excluding patients with an established high risk of bleeding or reduced life expectancy showed comparable discriminatory performance of the S_2_TOP-BLEED score 0.70 (0.64–0.77)

**Table 2.**
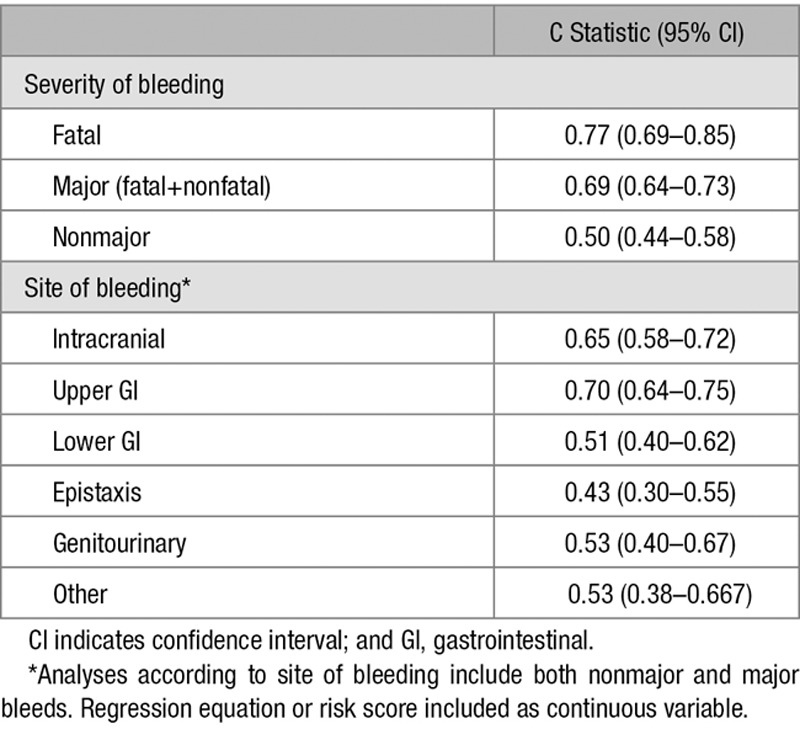
C Statistic (95% CI) of S_2_TOP-BLEED Score in Validation Cohort

The REACH score showed a C statistic of 0.63 (95% CI, 0.58–0.69) for major bleeding at 2 years and systematically underestimated risk of bleeding (Figure IA in the online-only Data Supplement). The Intracranial-B_2_LEED_3_S score had a C statistic of 0.60 (95% CI, 0.51–0.70) for intracranial bleeding at 2 years and showed accurate calibration (Figure IB in the online-only Data Supplement). The S_2_TOP-BLEED score showed improved reclassification and integrated discrimination as compared with the REACH and Intrancranial-B_2_LEED_3_S scores (Table V in the online-only Data Supplement).

A model with 5 age categories only as defined in the S_2_TOP-BLEED score (45–54, 55–64, 65–74, 75–85, and 85+) showed a C statistic of 0.66 (0.62–0.71), and a model containing 4 age categories as defined in the REACH score (45–54, 55–64, 65–74, and 75+) had a C statistic of 0.64 (0.60–0.69; Table VI in the online-only Data Supplement). The predictive performance of the models without age was 0.57 (0.51–0.64) for S_2_TOP-BLEED and 0.52 (0.45–0.58) for REACH.

**Figure 3.**
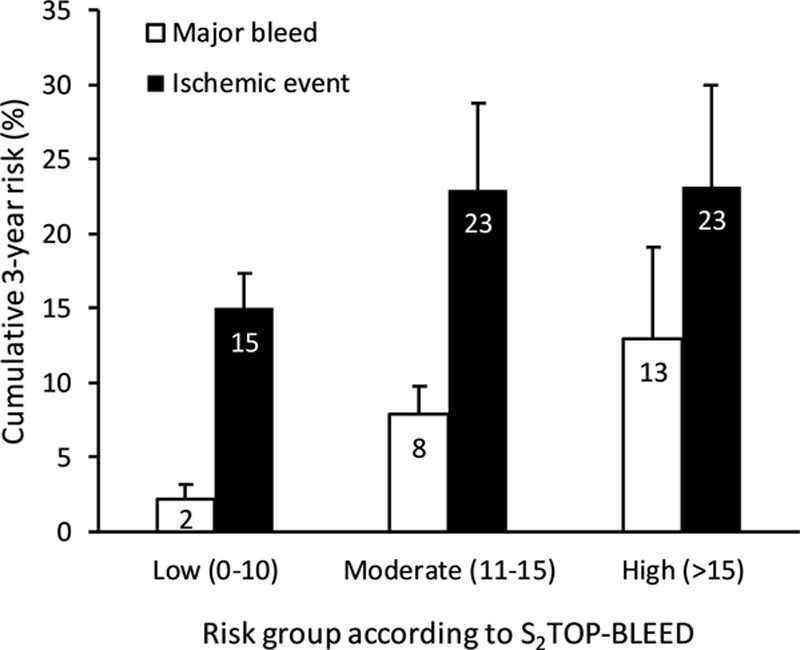
Cumulative 3-year risk of recurrent ischemic events and major bleeding events across risk groups of the S_2_TOP-BLEED score. Observed 3-year risk of major bleeds and recurrent ischemic events across predefined risk groups of the S_2_TOP-BLEED score.

Four hundred thirty-eight patients experienced a recurrent ischemic event during follow-up, and the overall observed 3-year risk was 19% (95% CI, 17%–21%). The C statistic of the S_2_TOP-BLEED score for predicting recurrent ischemic events was 0.58 (95% CI, 0.55–0.61). Three-year risk of recurrent ischemic events was 15% (95% CI, 12%–17%) in the low bleeding risk group and 23% (95% CI, 16%–30%) in both the medium- and high-risk group (Figure 3; *P* for trend =0.22). The ratio of ischemic events versus bleeds decreased from 7.5:1 in the low-risk group to 2.9:1 in the intermediate-risk group and 1.8:1 in the high-risk group (*P* for trend <0.001).

## Discussion

We externally validated the S_2_TOP-BLEED score for major bleeding in patients with a TIA or ischemic stroke in a population-based cohort and found modest discriminatory performance and calibration. Compared with the REACH and Intracranial-B_2_LEED_3_S scores, the S_2_TOP-BLEED score showed best performance, both for prediction of intracranial and major bleeds. Although high bleeding risks were also associated with high risks of recurrent ischemic events, risk stratification may still be useful to identify a group of patients at particularly high risk of bleeding, in whom preventive measures are indicated.

Discriminatory performance of the S_2_TOP-BLEED score slightly improved compared with the original development study (C statistic 0.69; 95% CI, 0.64–0.73 versus 0.63; 95% CI, 0.61–0.64). This is likely explained by the fact that the validation cohort is more heterogeneous than the development cohort, as patients were not selected on the basis of strict inclusion and exclusion criteria. In general, external validation studies tend to show a drop in performance of models, often because of overfitting of risk scores in the development data.^15,24^ The observation that performance is maintained in a broader setting underlines the robustness of the model and confirms its generalizability to a wide range of stroke patients. Also, performance of the model is maintained after excluding patients with an established high bleeding risk or reduced life expectancy, showing that the model can help to stratify patients in the group with most uncertainty about the risk of bleeding. Of note, the S_2_TOP-BLEED score performed particularly well for prediction of major and fatal bleeds, which are of clinical importance and may substantially offset the benefit of antiplatelet drugs.

The REACH score systematically underestimated risk of bleeding, which is likely because of the fact that the model was derived from patients with or at risk of atherothrombosis. It has been shown previously that patients with symptomatic vascular disease have higher risks of bleeding than patients with risk factors only.^25^ The slightly lower discriminatory performance of REACH compared with S_2_TOP-BLEED can partly be explained by differences in the representation of age in both models, as shown by differences in C statistics for models containing age only. In the REACH score, the weights assigned to age groups imply a linear association between age and bleeding, while the risk of bleeding tends to increase more rapidly at older ages.^5^ Also, the elderly patients were not represented separately in the REACH score (the highest category was >75 years), whereas nearly half of all patients with a TIA or stroke are over 75 years of age.^5^ Although age was the most important factor in predicting risk of bleeding, other variables in the S_2_TOP-BLEED score do have a relevant contribution to risk prediction, as is shown in Figure II in the online-only Data Supplement; younger patients with multiple risk factors may have higher predicted risk of bleeding than patients in older age groups without additional risk factors.

Although the C statistic improved slightly compared with the development cohort, values below 0.7 are still considered moderately discriminative. However, similar C statistics are seen for bleeding risk scores in other domains, such as for the HAS-BLED (hypertension, abnormal renal/liver function, stroke, bleeding history or predisposition, labile international normalized ratio, elderly, drugs/alcohol concomitantly) and ORBIT (older age, reduced haemoglobin/haematocrit/anaemia, bleeding history, insufficient kidney function, treatment with antiplatelets) scores in atrial fibrillation.^26,27^ Furthermore, calibration of a risk score is as important as its discrimination or may be considered even more important in the current setting, where risk of bleeding has to be weighed against the risk of recurrent ischemic events. We showed that long-term predicted risks accurately corresponded with observed risks. The fact that the model showed good calibration in the validation cohort despite differences in baseline risk and case-mix indicates that variables in the model accounted for most of the differences between the 2 cohorts.

As shown previously, high bleeding risks are associated with high risks of recurrent ischemic events.^28^ As such, high estimated bleeding risks cannot easily guide treatment decisions of antiplatelet therapy and should always be accompanied by the assessment of ischemic event risk. However, our results do show that risk of ischemic events stabilizes while risk of bleeding increases in patients in medium- and high-risk groups of the S_2_TOP-BLEED score. Risk stratification may therefore be useful to identify patients in the high-risk group in whom caution seems warranted before starting aggressive dual antiplatelet therapy. Also, estimation of bleeding risk may help to identify patients in whom gastroprotective agents might be indicated. Trials have shown that proton pump inhibitors (PPI) effectively reduce the risk of upper GI bleeding by 70% to 90%,^29^ but in clinical practice, proton pump inhibitors are not routinely prescribed, possibly because of concerns over side effects associated with long-term use.^30,31^ A recent study has shown that the numbers needed to treat to prevent one upper GI bleed in patients on aspirin are reasonable, particularly in elderly patients (numbers needed to treat 23 to prevent one upper GI bleed at 5 years in patients aged ≥75 years).^5^ Co-prescription of proton pump inhibitors may be an effective intervention to lower the risk of GI bleeds, but safety of long-term proton pump inhibitor treatment has not been established in a randomized trial yet. Furthermore, high predicted bleeding risks may trigger physicians to treat and monitor hypertension more closely, aiming to reduce risk of intracerebral hemorrhages.^32^

Strengths of our study include the population-based nature of the study, the thorough ascertainment of bleeding events through multiple overlapping sources and the long-term follow-up. However, there are also some limitations. Not all variables included in the risk scores were available in the validation cohort, but suitable proxies could be found for most variables. Furthermore, the number of bleeds in the validation cohort was moderate, particularly for the assessment of performance according to site and severity. Last, a small proportion of patients were excluded as they were not prescribed antiplatelet drugs because of recent bleeding or intolerance. However, this reflects clinical practice.

In conclusion, the current study shows that the S_2_TOP-BLEED score can be used to estimate the risk of major bleeding in patients with a TIA or ischemic stroke on antiplatelet drugs. Although the risk of recurrent ischemic events will outweigh the risk of bleeding in the majority of patients, the risk score identifies patients at particularly high risk of bleeding in whom preventive measures should be taken. Future studies may focus on refinement of the S_2_TOP-BLEED score for major bleeding by including results from laboratory tests, such as renal failure and anemia, or radiological characteristics, such as microbleeds. Also, a more thorough assessment of the balance between benefits and risks of long-term antiplatelet drugs is required, incorporating risk estimates on risk of recurrent ischemic events, as well as risk of bleeding.

## Acknowledgments

We are grateful to all the staff in the general practices that collaborated in OXVASC (Oxford Vascular Study): Abingdon Surgery, Stert St, Abingdon; Malthouse Surgery, Abingdon; Marcham Road Family Health Centre, Abingdon; The Health Centre, Berinsfield; Key Medical Practice; Kidlington; 19 Beaumont St, Oxford; East Oxford Health Centre, Oxford; Church Street Practice, Wantage. We also acknowledge the use of the facilities of the Acute Vascular Imaging Centre, Oxford.

## Sources of Funding

The Oxford Vascular Study is funded by the National Institute for Health Research (NIHR) Oxford Biomedical Research Centre (BRC), Wellcome Trust, Wolfson Foundation, and British Heart Foundation. Dr Rothwell is in receipt of a NIHR Senior Investigator award. Drs Greving and Hilkens are supported by a grant from the Dutch Heart Foundation (grant number 2013T128). The views expressed are those of the author(s) and not necessarily those of the National Health Service, the NIHR, or the Department of Health.

## Disclosures

None.

## Supplementary Material
