# Supplementary material for: External Validation of Risk Scores for Major Bleeding in a Population-Based Cohort of Transient Ischemic Attack and Ischemic Stroke Patients
Source: Stroke. 2018 Feb 19;49(3):601–6. doi: 10.1161/STROKEAHA.117.019259 (PMC5839707; doi:10.1161/STROKEAHA.117.019259)
Supplement: Supplementary file 1 [file str-49-601-s001.pdf]

External validation of risk scores for major bleeding in a population-based cohort of TIA and ischemic stroke patients

Nina A Hilkens, MD, Linxin Li, MD, DPhil, Peter M Rothwell, MD, PhD, FRCP, FMedSci, Ale Algra, MD, PhD, Jacoba P Greving, PhD

SUPPLEMENTAL MATERIAL

Content

Table I. Characteristics of development cohort (CAT) and validation cohort (OXVASC)

Table II. Score chart of the S_2TOP-BLEED score

Table III. Overview of REACH, Intracranial-B_2LEED_3S and S_2TOP-BLEED scores

Table IV. Site and severity of bleeds in OXVASC

Table V: Comparison of S_2TOP-BLEED, REACH and Intracranial-BLEEDS scores

Table VI. Representation of age in risk scores for major bleeding and their performance

Figure I. Calibration of REACH risk score and Intracranial-B_2LEED_3S score

Figure II. Predicted three-year risk of major bleeding according to the S_2TOP-BLEED score per age group

Table I. Characteristics of development cohort (CAT) and validation cohort (OXVASC)

	CAT	OXVASC
**Source**	Trials: CAPRIE, ESPS-2, MATCH, CHARISMA, ESPRIT, PRoFESS	Population-based cohort
**Inclusion criteria**	TIA or non-cardioembolic ischemic stroke	TIA or ischemic stroke
**Inclusion period**	1989-2006	2002-2012
**Sample size**	43,112	2,072
**Region**	North-America, South-America, Europe, Asia, Australia, South-Africa	Oxfordshire, UK
**Outcome**	Trial specific definitions of major bleeding	CURE criteria for bleeding
**Follow-up, years (median, range)**	2.0 (0-8.1)	3.7 (0-10.9)
**No of major bleeds**	1,530	117

Table II. Score chart of S_2TOP-BLEED score for major bleeding[1]

**Factor**	**Points**
**S**ex - male	2
**S**moking - current	1
**T**ype of antiplatelet agent	
Clopidogrel	0
Aspirin (+/- Dipyridamole)	1
Aspirin-Clopidogrel	5
**O**utcome on mRS 3-5	2
**P**rior stroke	1
**B**lood pressure (hypertension)	1
**L**ow BMI	
<20	2
20-25	1
>25	0
**E**lderly	
45-55	2
55-65	4
65-75	6
75-85	9
>85	12
**E**thnicity - Asian	1
**D**iabetes	1

mRS: modified Rankin Scale

Table III. Overview of REACH, Intracranial-B_2LEED_3S and S_2TOP-BLEED scores

Risk score	Study population	Development cohort	Number of bleeds /N	Outcome	Prediction horizon	Included items	Performance in development cohort
**REACH**[2]	Patients with or at risk of atherothrombosis	REACH registry	804/ 56,616	Major bleeding	2 year	Age<br>Smoking<br>Hypertension<br>Diabetes<br>Hypercholesterolemia<br>Peripheral arterial disease<br>Heart failure<br>Antiplatelet agents<br>Oral anticoagulants	0.68
**Intracranial B_2LEED_3S**[3]	Patients with TIA or non-cardioembolic ischemic stroke	PERFORM trial	263/ 19,100	Intracranial bleeding	2 year	Age<br>Sex<br>BMI<br>Asian ethnicity<br>Hypertension<br>Cardiovascular disease<br>Cerebrovascular disease<br>Lacune/small vessel disease<br>Dual antiplatelet or oral anticoagulants	0.64 (0.61-0.67)
**S_2TOP-BLEED**[1]	Patients with TIA or non-cardioembolic ischemic stroke	CAPRIE, ESPS 2, MATCH, CHARISMA, ESPRIT, PRoFESS trials	1530/ 43,112	Major bleeding	3 year	Age<br>Sex<br>BMI<br>Asian ethnicity<br>Smoking<br>Hypertension<br>Diabetes<br>Stroke<br>mRS score<br>Antiplatelet agents	0.63 (0.61-0.64)

Table IV. Site and severity of bleeding in OXVASC

	Severity of bleeding				
	**Non-major, N**	**Major, N**	**Life-threatening, N**	**Fatal, N**	**Total, N (%)**
Intracranial	0	0	17	19	**36 (14%)**
Upper GI	35	16	20	10	**81 (32%)**
Lower GI	26	6	4	1	**37 (15%)**
Unknown GI	5	3	4	1	**13 (5%)**
Epistaxis	29	1	1	0	**31 (12%)**
Genitourinary	29	4	2	0	**35 (14%)**
Other	13	4	4	0	**21 (8%)**
**Total**	**137 (54)**	**34 (13)**	**52 (20)**	**31 (12)**	**254 (100%)**

GI: gastro-intestinal

Table V: Comparison of S_2TOP-BLEED, REACH and Intracranial-BLEEDS scores

	Major bleeding			Intracranial bleeding		
	**C-statistic (95% CI)**	**NRI**	**IDI**	**C-statistic (95% CI)**	**NRI**	**IDI**
S_2TOP-BLEED	0.69 (0.63-0.73)	0.27	0.006	0.65 (0.60-0.73)	0.39	0.0004
REACH	0.63 (0.58-0.69)	Ref	Ref	-	-	-
Intracranial B_2LEED_2S	-	-	-	0.60 (0.51-0.70)	Ref	Ref

CI: confidence interval; NRI: net reclassification improvement; IDI: integrated discrimination improvement; Ref: reference

Table VI. Representation of age in S_2TOP-BLEED and REACH scores and their performance

Risk score	Age groups	Points	C-statistic – age only	C-statistic – overall
**S_2TOP-BLEED**			0.66 (0.61-0.71)	0.69 (0.65-0.74)
	45-54	2		
	55-64	4		
	65-74	6		
	75-84	9		
	≥85	12		
**REACH**			0.64 (0.60-0.69)	0.63 (0.58-0.69)
	45-54	0		
	55-64	2		
	65-74	4		
	≥75	6		

Figure I. Calibration of REACH score and Intracranial B_2LEED_3S score

A **B**

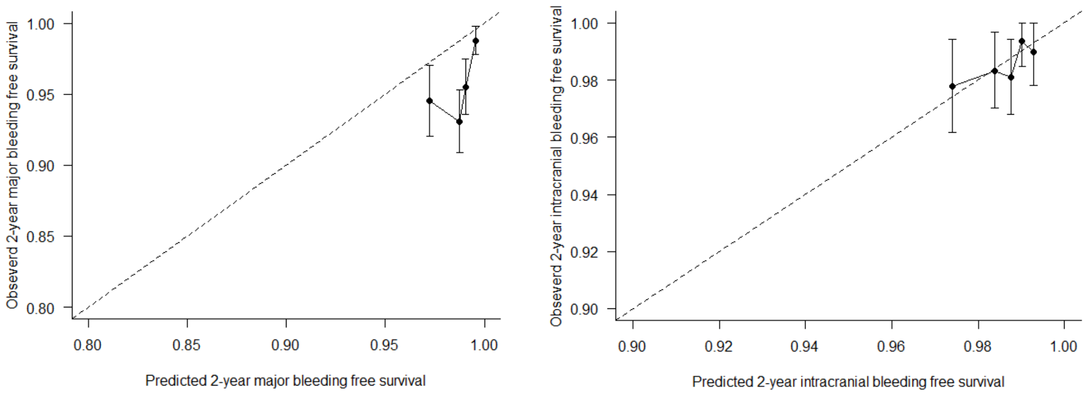

Figure Legend: Calibration of REACH score for two-year risk of major bleeding in OXVASC (A) and calibration of Intracranial-B_2LEED_3S score for two-year risk of intracranial hemorrhage in OXVASC (B)

Figure II. Predicted three-year risk of major bleeding according to the S_2TOP-BLEED score per age group

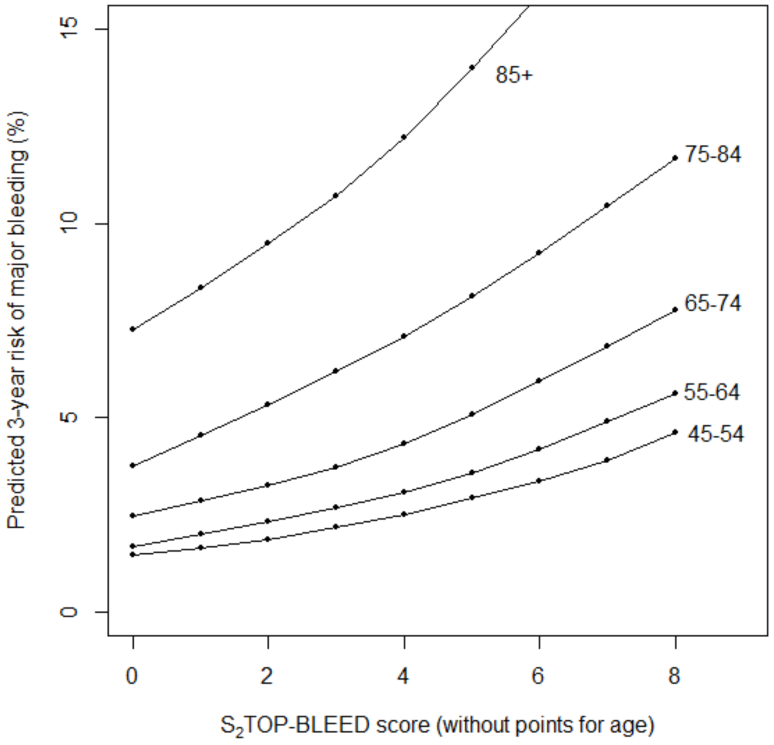

References

1. Hilkens NA, Algra A, Diener HC, Reitsma JB, Bath PM, Csiba L, et al. Predicting major bleeding in patients with noncardioembolic stroke on antiplatelets: S2TOP-BLEED. *Neurology*. 2017;89:936-943.

2. Ducrocq G, Wallace JS, Baron G, Ravaud P, Alberts MJ, Wilson PW, et al. Risk score to predict serious bleeding in stable outpatients with or at risk of atherothrombosis. *Eur Heart J*. 2010;31:1257-1265.

3. Amarenco P, Sissani L, Labreuche J, Vicaut E, Bousser MG, Chamorro A, et al. The intracranial-B2LEED3S score and the risk of intracranial hemorrhage in ischemic stroke patients under antiplatelet treatment. *Cerebrovasc Dis*. 2017;43:145-151.
